# LsrR-Mediated Quorum Sensing Controls Invasiveness of *Salmonella typhimurium* by Regulating SPI-1 and Flagella Genes

**DOI:** 10.1371/journal.pone.0037059

**Published:** 2012-05-18

**Authors:** Jeongjoon Choi, Dongwoo Shin, Minjeong Kim, Joowon Park, Sangyong Lim, Sangryeol Ryu

**Affiliations:** 1 Department of Food and Animal Biotechnology, Department of Agricultural Biotechnology, Center for Agricultural Biomaterials, and Research Institute for Agriculture and Life Sciences, Seoul National University, Seoul, Korea; 2 Department of Molecular Cell Biology, Samsung Biomedical Research Institute, Sungkyunkwan University School of Medicine, Suwon, Korea; 3 Radiation Research Division for Biotechnology, Korea Atomic Energy Research Institute, Jeongeup, Korea; Wadsworth Center, New York State Department of Health, United States of America

## Abstract

Bacterial cell-to-cell communication, termed quorum sensing (QS), controls bacterial behavior by using various signal molecules. Despite the fact that the LuxS/autoinducer-2 (AI-2) QS system is necessary for normal expression of *Salmonella* pathogenicity island-1 (SPI-1), the mechanism remains unknown. Here, we report that the LsrR protein, a transcriptional regulator known to be involved in LuxS/AI-2-mediated QS, is also associated with the regulation of SPI-1-mediated *Salmonella* virulence. We determined that LsrR negatively controls SPI-1 and flagella gene expressions. As phosphorylated AI-2 binds to and inactivates LsrR, LsrR remains active and decreases expression of SPI-1 and flagella genes in the *luxS* mutant. The reduced expression of those genes resulted in impaired invasion of *Salmonella* into epithelial cells. Expression of SPI-1 and flagella genes was also reduced by overexpression of the LsrR regulator from a plasmid, but was relieved by exogenous AI-2, which binds to and inactivates LsrR. These results imply that LsrR plays an important role in selecting infectious niche of *Salmonella* in QS dependent mode.

## Introduction

Bacteria control gene expression patterns in response to changes in their population density through a process called quorum sensing (QS). In QS, small signaling molecules called autoinducers are synthesized and released from the bacterial cells, and accumulate in the external environment [1]. When cell density reaches a certain level, producing autoinducer concentrations over a minimal threshold, the autoinducers bind to cognate receptors to promote changes in gene expression [2]. QS systems regulate a large number of physiological processes in bacteria including biofilm formation, virulence factor production, bioluminescence, sporulation, motility, and antibiotic production [3], [4], [5], [6], [7].

Several types of QS systems have been described in various species of bacteria. Many Gram-negative bacteria employ acyl homoserine lactones for intraspecies communication [8], whereas Gram-positive autoinducers are typically peptides [9]. Another QS pathway, in addition, in which signaling is mediated by the LuxS-produced autoinducer-2 (AI-2), has been found in both Gram-negative and Gram-positive bacteria [10]. The LuxS protein is found in over 55 bacterial species [11], and catalyzes the conversion of S-ribosylhomocysteine to 4,5-dihydroxy-2,3-pentanedione (DPD), which spontaneously cyclizes to form the signaling molecule AI-2 [10].

The LuxS/AI-2 QS system is present in *Salmonella enterica* serovar Typhimurium (*S*. Typhimurium), whose signal molecule has been identified as (2R,4S)-2-methyl-2,3,3,4-tetrahydroxytetrahydrofuran [12]. In *S*. Typhimurium, AI-2 is imported by the Lsr transporter, an adenosine triphosphate-binding-cassette transporter encoded by the *lsr* operon and comprised of LsrA, LsrB, LsrC, and LsrD. The internalized AI-2 is phosphorylated by LsrK and modified further by LsrF and LsrG [13]. Phosphorylated AI-2 (phospho-AI-2) binds to the repressor protein LsrR, inactivating it and derepressing transcription of the *lsr* operon (see Fig. S1) [13].

*S.* Typhimurium causes gastroenteritis and diarrhea in humans due to acute intestinal inflammation, and also causes a typhoid-like disease in mice. During infection of animal hosts, *Salmonella* invade epithelial cells of the small intestine. Invasion is mediated by a type three secretion system encoded in *Salmonella* pathogenicity island 1 (SPI-1). The SPI-1 type three secretion system forms a needlelike complex through which a number of effector proteins are translocated into host cells and mediate modification of the actin cytoskeleton [14]. Expression of SPI-1 is controlled by various environmental cues including oxygen tension, osmolarity, pH, and nutrients, reflecting the complex conditions in the intestinal lumen [15], [16], [17].

Previously, we reported that LuxS-mediated QS is required for normal expression of a subset of genes within SPI-1 and contributes to virulence of *S.* Typhimurium because deletion of the *luxS* gene decreased the transcription of SPI-1 genes and impaired invasion of *Salmonella* [18]. In contrast, another study recently reported that mutation of the *luxS* gene has no effects on the expression of SPI-1 and *Salmonella* virulence in a mouse infection model [19].

LuxS could have different effects on SPI-1 expression depending on the exact experimental conditions because of its pleiotropic functions [20]. Thus, the contradictory results may be resolved by a more complete understanding of LuxS effects on SPI-1 expression. We sought to clarify the role of LuxS by finding a factor that links LuxS/AI-2-mediated QS and SPI-1 expression. In the present study, we report that LsrR, a DNA-binding repressor of the *lsr* operon [21], negatively controls expression of SPI-1 and flagella genes and also regulates the ability of *Salmonella* to invade host cells.

## Results

### *Salmonella LuxS* Mutant is Indeed Defective for InvF-dependent Expression of SPI-1 Genes and Attenuated for Virulence in Mice

*luxS* deletion mutants have widely been used to reveal the roles of QS in bacterial physiology. However, the role of LuxS in *Salmonella* pathogenesis is controversial. We reported that a *Salmonella* strain lacking the *luxS* gene displays defects in virulence phenotypes associated with SPI-1 expression [18], but these findings were not reproduced by others [19]. These contradictory results could be due to the pleiotropic effects of *luxS* mutation [20], or reflect a recently described non-quorum sensing function of LuxS [22]. To clarify the role of LuxS in *Salmonella* virulence, it is necessary to identify the factor that directly connects LuxS-dependent QS to regulation of SPI-1 virulence genes.

**Figure 1:**
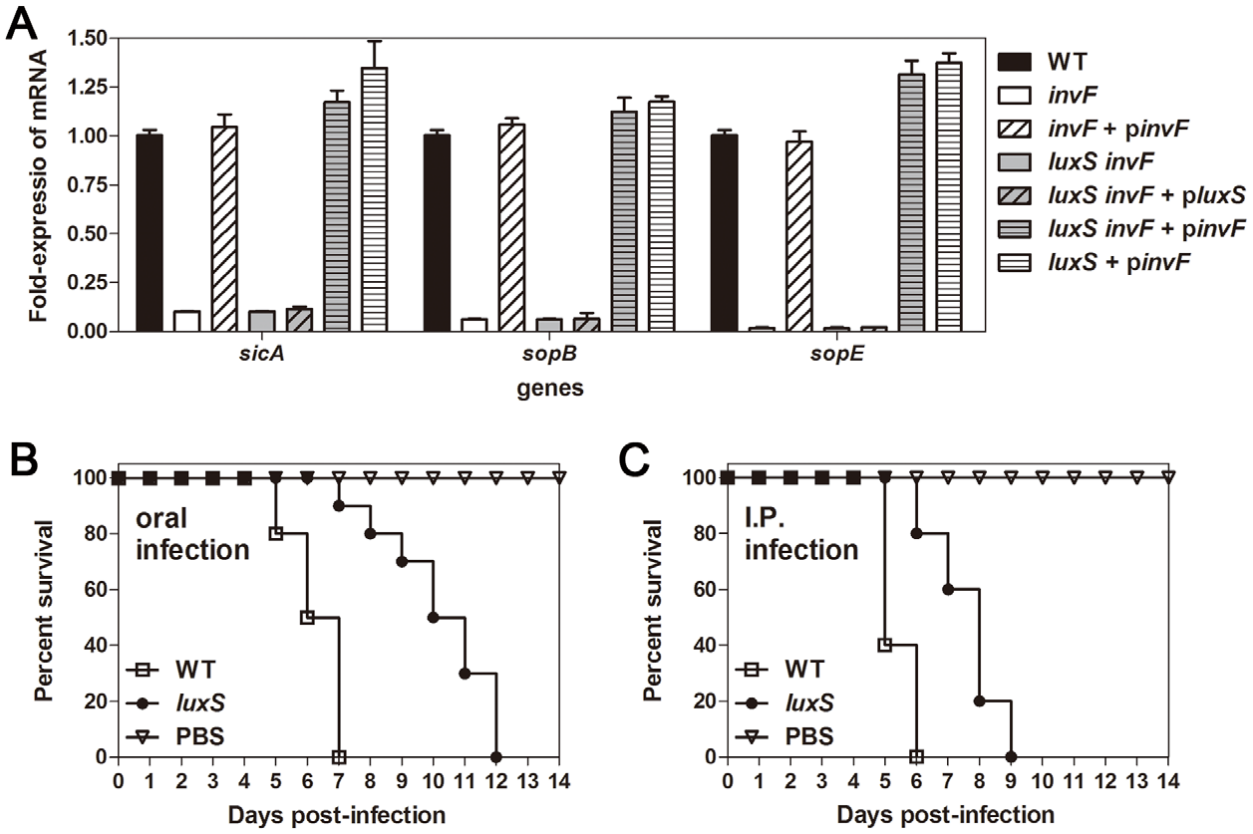
The *luxS* gene is necessary for normal expression of SPI-1 and virulence. (A) Transcriptional levels of the SPI-1 genes *sicA, sopB,* and *sopE* were determined by qRT-PCR. Overnight cultures of wild-type (WT) and mutant strains were diluted in fresh LB and mRNA samples were prepared from stationary phase of static cultures. Values are means and standard deviations of three independent experiments. (B and C) Six-week-old BALB/c mice (n = 10) were infected orally with 10^7^ CFU (B) or intraperitoneally (I.P.) with 10^3^ CFU (C) *Salmonella* strains. Mice surviving after infection were monitored daily for two weeks.

Before searching for such a factor, we confirmed that *luxS* is required for normal SPI-1-mediated *Salmonella* virulence. We showed that *luxS* deletion causes reduced expression of the *invF* gene, which in turn decreases expression of the InvF-regulated SPI-1 genes *sicA*, *sigD*, and *sopE* [18]. By conducting qRT-PCR, we determined that the mRNA levels of the *sicA, sopB,* and *sopE* genes decrease in both the Δ*invF* strain and the Δ*luxS* Δ*invF* double mutant strain (Fig. 1A). Expression of *invF* from a plasmid restored transcription of *sicA*, *sopB* and *sopE* to wild-type levels in the Δ*luxS* Δ*invF* and the Δ*luxS* strains as well as the *invF* deletion strain (Fig. 1A). In contrast, the *luxS* expression from a plasmid failed to do so in both the Δ*luxS* Δ*invF* and the Δ*invF* strains (Fig. 1A and data not shown). These data indicate that the reduced transcription levels of a subset of SPI-1 genes in the *luxS* deletion mutant were due to a decrease in InvF levels.

Our previous finding showed that the *luxS* mutant is attenuated for virulence in mice was based on bacterial numbers in liver and spleen [18], whereas Perrett *et al.* [19] reported that the *luxS* mutant did not display any significant differences in virulence compared to wild-type was based on number of live mice at day 12 post infection [19]. We decided to re-evaluate the virulence of the *luxS* mutant strain with a detailed monitoring (i.e. monitoring for every 12 or 24 h) of the survival of mice infected with *Salmonella*. Our data also show that all mice infected with either the wild-type or the *luxS* mutant strain died by 12 days after oral or intraperitoneal challenge (Fig. 1B and C), but the survival of mice infected with the *luxS* mutant was significantly delayed compared to those infected with wild-type *Salmonella* (Fig. 1B and C, *p*<0.0001). Taken together, these data emphasize that LuxS is necessary for normal expression of SPI-1 by controlling InvF and also for normal virulence of *Salmonella.*

### LsrR Negatively Controls Expression of SPI-1 Genes in the Absence of LuxS

Because LuxS is not a DNA-binding protein, we hypothesize that there must be a transcription factor that links QS and SPI-1 regulation as described in other bacteria that regulate gene expression in response to QS [11]. In this sense, we focused on the LsrR protein, the repressor of the *lsr* operon, because it is the only known regulator controlled by *Salmonella* QS [1].

**Figure 2:**
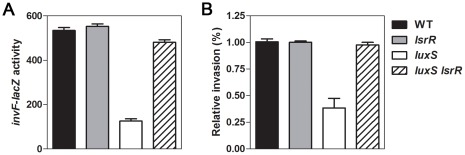
LsrR is involved in the regulation of SPI-1 expression and invasion of *S. typhimurium*. (A) Wild-type (WT) and mutant strains carrying a chromosomal *invF-lacZ* fusion were diluted in LB medium and β-galactosidase activity (Miller units) was determined at 4 h of cultures grown with shaking. Values are the means and standard deviation of three independent experiments. (B) Monolayers of HEp-2 epithelial cells were infected with the wild-type (WT) and mutant strains and numbers of internalized bacteria were determined (see Methods). Values represent the relative amount of internalized bacteria normalized to the level of internalization of the WT strain, which was set to 1.00. Values are the average and standard deviation from three independent experiments, each done in triplicate.

Phospho-AI-2 binds to LsrR inactivating its ability to act as a repressor and thereby inducing expression of the *lsr* operon [13]. Because the *luxS* deletion mutant cannot produce AI-2, LsrR is constitutively active. If LsrR negatively regulates the expression of SPI-1 genes, then deletion of the *luxS* gene would be expected to decrease expression of SPI-1 genes as we reported previously [18]. To test the hypothesis that LsrR lowers expression of SPI-1 genes, we constructed Δ*lsrR* and Δ*lsrR* Δ*luxS* strains carrying a chromosomal *invF-lacZ* fusion. β-galactosidase assay determined that deletion of *lsrR* had no effect on transcription of the *invF-lacZ* fusion (Fig. 2A). However, the absence of LsrR compensated for the transcriptional defect in *invF-lacZ* expression in the *luxS* deletion mutant (Fig. 2A). The regulatory effects of LsrR in the absence of *luxS* gene were analyzed in cultures with (Fig.2A) or without shaking (data not shown). This result was confirmed to be due to the absence of LsrR function because expression of LsrR from a heterologous promoter reduced *invF* transcription in the Δ*luxS* Δ*lsrR* double mutant strain (Fig. S2). These results suggest that in the absence of LuxS-catalyzed AI-2 production, LsrR represses InvF expression, which in turn reduces transcription levels of the InvF-regulated genes within SPI-1.

The internalization of *Salmonella* into epithelial tissues of animal hosts is mediated by a number of gene products expressed from the SPI-1 locus [14], suggesting a role for LsrR in regulating internalization. We next examined whether LsrR regulates the invasiveness of *Salmonella*. Deletion of *luxS* impaired the ability of *Salmonella* to invade HEp-2 epithelial cells (Fig. 2B), as we reported previously [18]. If both *lsrR* and *luxS* are deleted, however, invasion of HEp-2 cells is comparable to that of the parental *lsrR* ^+^ *luxS* ^+^ strain (Fig. 2B). These results are in good agreement with the finding that *lsrR* deletion restored expression levels of InvF in the *luxS* deletion strain (Fig. 2A). Taken together, our results suggest that, when activated by the absence of AI-2, the LsrR protein represses SPI-1 expression, which impairs *Salmonella*’s invasiveness.

### Overexpression of the LsrR Protein Lowers Expression of SPI-1 Genes

The down-regulation of *invF* expression by LsrR was only observed in the absence of LuxS (Fig. 2). This may be because the levels of active LsrR protein (i.e., LsrR not associated with AI-2) were elevated in the absence of LuxS. In contrast, LsrR could be present in a phospho-AI-2-bound inactive form in a *luxS* ^+^ strain, which could explain why deletion of *lsrR* gene alone did not alter *invF* expression (Fig. 2A). If this were the case, overexpression of the LsrR protein could increase levels of active LsrR protein to a level sufficient to suppress expression of SPI-1 genes even in a strain carrying the *luxS* gene.

**Figure 3:**
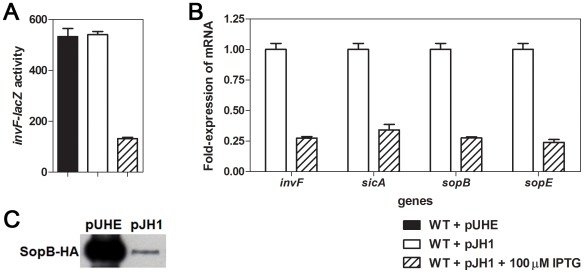
LsrR negatively controls the expression of SPI-1 genes. (A) Wild-type (WT) *Salmonella* carrying a chromosomal *invF-lacZ* fusion and either the control plasmid, pUHE21-2*lacI^q^* or the *lasR* ^+^ plasmid pJH1 were grown in LB with shaking for 4 h. Production of LsrR was induced by the addition of 100 µM IPTG. (B) WT *Salmonella* carrying pJH1 were grown LB or LB supplemented with 100 µM IPTG, with shaking for 4 h. The mRNA levels of SPI-1 genes were determined by qRT-PCR. Shown in (A) and (B) are the mean values and standard deviation of three independent experiments. (C) Western blot analysis was conducted on cell extracts prepared from the strain harboring either pUHE21-2*lacI^q^* or pJH1 grown in LB with 100 µM IPTG, with shaking for 4 h. These strains express the SopB protein tagged with a HA-epitope from the normal chromosomal location.

To test this idea, we constructed a strain carrying a chromosomal *invF-lacZ* fusion and harboring the plasmid pJH1 in which expression of LsrR is under control of the *lac* promoter. β-galactosidase assays show that IPTG induction of LsrR protein lowers the transcription levels of *invF* by about 4-fold (Fig. 3A). qRT-PCR analysis confirms that overexpression of LsrR decreases transcription of several SPI-1 genes: the transcript levels of the *invF*, *sicA*, *sopB*, and *sopE* genes were reduced by approximately 3- to 4-fold when the wild-type strain harboring pJH1 was grown with IPTG (Fig. 3B). Next, we used Western blot analysis to investigate whether levels of a SPI-1 protein were affected by LsrR overexpression. Western blot analysis determined that overexpression of LsrR in the wild-type strain dramatically reduced levels of the SopB protein (Fig. 3C). Although the mRNA fold changes and protein levels were different in the LsrR-overexpression and wild-type strains, the negative regulatory effects of LsrR were consistent. The data presented here demonstrate that the LsrR regulator negatively controls expression of SPI-1 genes.

### Overexpression of LsrR Inhibits Invasion into Epithelial Cells

**Figure 4:**
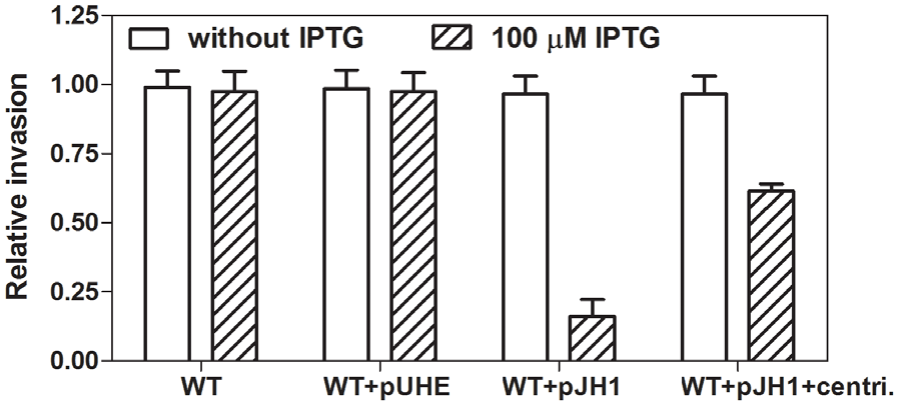
The overexpression of LsrR decreased *Salmonella* invasion into HEp-2 epithelial cells, even when the requirement for motility is bypassed through centrifugation. Monolayers of HEp-2 epithelial cells were infected with the wild-type (WT) *Salmonella*, WT harboring backbone plasmid, pUHE21-2*lacI^q^*, and WT harboring pJH1 strains in the presence or absence of 100 µM IPTG. To exclude the requirement of motility, mild centrifugation was employed (centri.). The numbers of internalized bacteria were determined as described in Methods. Values represent the relative amount of internalized bacteria and have been normalized to the level of internalization of WT strain, which was set at 1.00. Values are the average and standard deviation from three independent experiments, each done in triplicate.

We next investigated whether the reduced expression of SPI-1 genes resulting from LsrR overexpression affects the ability of *Salmonella* to invade mammalian cells. When HEp-2 epithelial cells were incubated with the wild-type strain carrying pJH1 that had been grown with IPTG to induce LsrR expression, invasion was greatly impaired (i.e., 17% invasion compared to that of the wild-type strain; Fig. 4).

Expression of SPI-1 and motility are both required for *Salmonella* cells to gain entry into epithelial cells [14], [23], but the requirement for motility can be overcome through the use of mild centrifugation [23]. To examine whether the defective invasion phenotype of LsrR-overexpressing *Salmonella* was solely due to the decrease in SPI-1 expression, or whether motility might also be affected, a mild centrifugation was applied to promote bacterium-host cell contacts during the invasion assay [23]. Although invasion was still decreased in the LsrR overexpressing strain when adherence of *Salmonella* cells to the epithelial cells was promoted using centrifugation (Fig. 4), invasion was only 62% that of the wild-type strain (Fig. 4). Thus, centrifugation partially restored invasion of HEp-2 cells by *Salmonella* overexpressing LsrR.

### The LsrR Protein Negatively Regulates Expression of Flagella Genes

**Figure 5:**
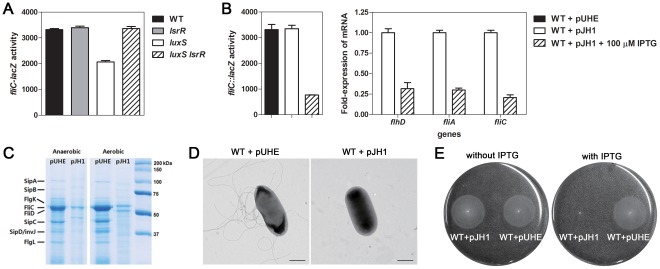
LsrR negatively controls the expression of flagella. (A) Wild-type (WT) and mutant strains carrying *fliC-lacZ* fusion on chromosome were diluted in LB medium grown with shaking, and β-galactosidase activity (Miller units) was determined at 4 h. Values are the means and standard deviation of three independent experiments. (B, left) Wild-type (WT) *Salmonella* carrying a chromosomal *invF-lacZ* fusion and either the control plasmid, pUHE21-2*lacI^q^* or the *lasR* ^+^ plasmid pJH1 were grown in LB with shaking for 4 h. Production of LsrR was induced by addition of 100 µM IPTG. (B, right) WT strain carrying pJH1 was grown in LB or LB with 100 µM IPTG, with shaking for 4 h. The mRNA levels of flagella genes were determined by qRT-PCR. Values are means and standard deviations of three independent experiments. (C) Representative SDS-PAGE gel of secreted proteins. Overnight cultures of the WT strain harboring either pUHE21-2*lacI^q^* or pJH1 were diluted (1∶100) into fresh LB broth in the presence or absence of 100 µM IPTG and grown for 4 h with (aerobic) or without (anaerobic) shaking. Secreted proteins were recovered from cell-free spent culture media by TCA precipitation. (D) Electron microscopic observation of flagella using negative stain. Samples were prepared from WT cells harboring either pUHE21-2*lacI^q^* or pJH1 grown in LB with 100 µM IPTG. The scale bar indicates 0.5 µm. (E) Phenotypic assay for motility was performed to confirm the down-regulation of flagella genes in LsrR-overexpressing *Salmonella* cells. A 1 µl aliquot of washed WT cells harboring either pUHE21-2*lacI^q^* or pJH1 was stab inoculated into 0.3% LB agar with or without 100 µM IPTG. The images were taken following 8 h of growth at 37°C.

Because the enhanced contact of bacterium-host cell by centrifugation partially recovered the defective invasiveness of LsrR-overexpressing *Salmonella*, we reasoned that motility of *Salmonella* might also be regulated by LsrR or LuxS. We examined the effect of *luxS* and *lsrR* deletions on expression of *fliC* which encodes flagellin, the major component of the flagellum [24], by using chromosomal *fliC-lacZ* fusion strains. β-galactosidase assays determined that *fliC* expression decreased in the *luxS* deletion mutant, unaffected by deletion of *lsrR*, and elevated in the Δ*luxS* Δ*lsrR* double mutant (Fig. 5A). These results indicate that LsrR represses transcription of the *fliC* gene in the absence of LuxS.

We next investigated the effect of LsrR overexpression on flagella gene expression. β-galactosidase assays revealed that in the *fliC-lacZ* fusion strain carrying pJH1, the transcription levels of *fliC* decreased by approximately 4-fold upon IPTG addition (Fig. 5B). Moreover, qRT-PCR determined that the mRNA levels of the *flhD* and *fliA*, which encode key regulators of flagella synthesis [24], were reduced by approximately 3-fold when the wild-type strain harboring pJH1 was grown with IPTG (Fig. 3D). Analysis of secreted proteins showed that LsrR overexpression in wild-type *Salmonella* greatly reduced levels of the flagella proteins, FliC, FliD, and FlgL (Fig. 5C). Levels of the SPI-1 effector proteins SipA, SipC, and SipD, were also lowered upon LsrR overexpression, consistent with the regulatory role of LsrR for SPI-1 genes (Fig. 5C). These results indicate that, in addition to regulating the SPI-1 genes, the LsrR protein also negatively regulates flagella genes.

### The LsrR Protein Suppresses Motility of *Salmonella*

Because expression levels of flagella decreased in the LsrR-overexpressing *Salmonella* strain (Fig. 5B and C), we asked whether the flagella structure is affected by LsrR levels. Transmission electron microscopy using a negative stain allows visualization of flagella appendages on the surface of the *S.* Typhimurium cells [24]. Using transmission electron microscopy, we were able to observe flagella on the *Salmonella* cells harboring the vector pUHE21-2*lacI^q^* (Fig. 5D), but not on cells carrying pJH1 grown in the presence of IPTG to induce LsrR overexpression (Fig. 5D).

We also compared the motility phenotypes of the wild-type *Salmonella* strains harboring pUHE21-2*lacI^q^* and pJH1. We found that both strains were motile in the absence of IPTG; however, motility of the strain carrying pJH1 was abolished in the presence of IPTG (Fig. 5E). These results show that the overexpression of LsrR suppresses *Salmonella* motility.

### LsrR-mediated QS Controls Expression of SPI-1 and Flagella Genes

**Figure 6:**
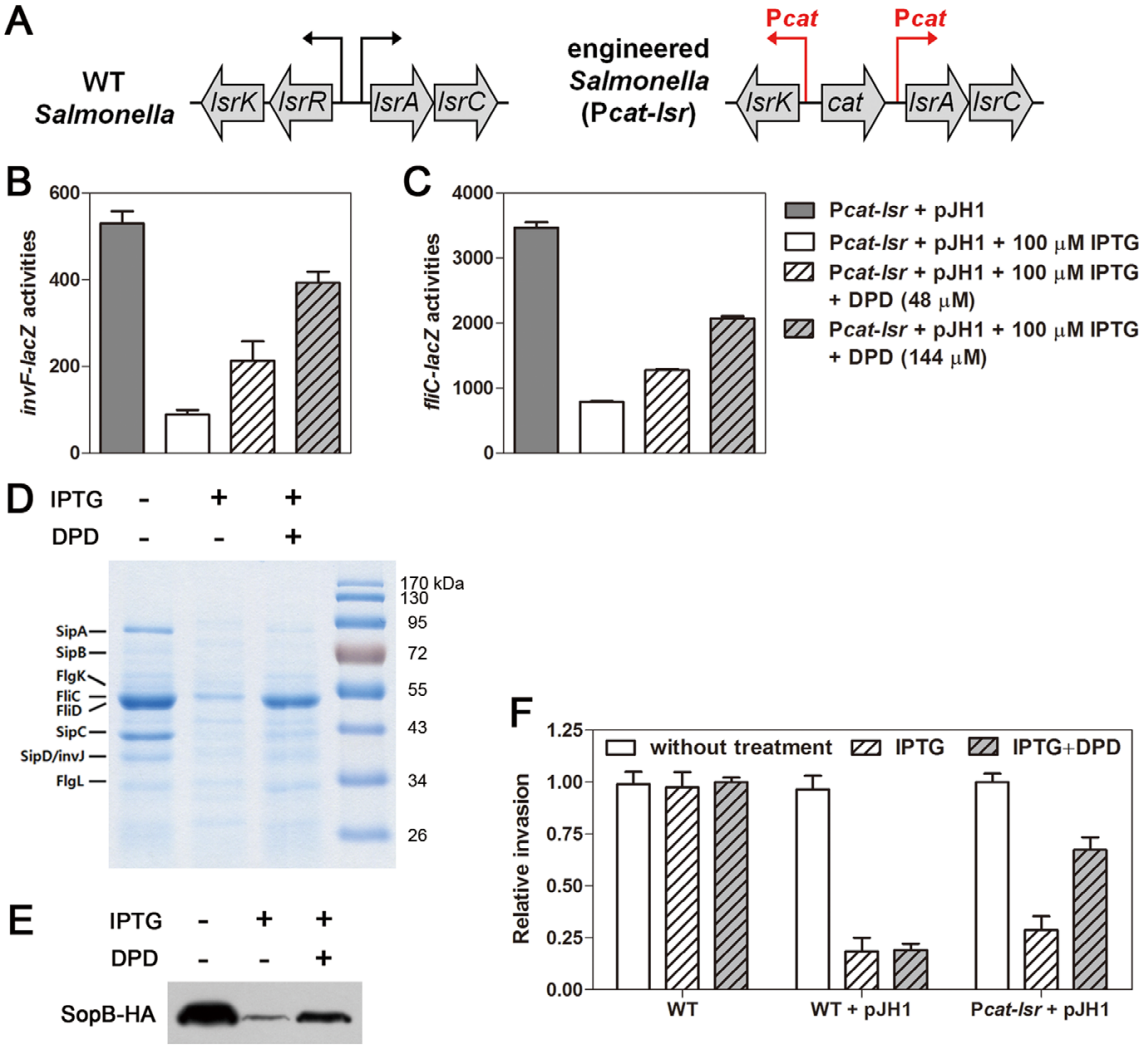
The regulatory function of LsrR was restored by exogenous autoinducer-2. (A) Schematic of the genomic context of the part of *lsr* operon, and the *lsrR*, and *lsrK* loci in the wild-type (WT) and P*cat-lsr* strains. The promoters of the *lsr* operon (*lsrA*) and *lsrK* have been replaced with the constitutively expressed promoter of the chloramphenicol resistant gene (P*cat*). (B and C) The P*cat*-*lsr* strains harboring pJH1 carrying a chromosomal *invF-lacZ* (B) or *fliC-lacZ* (C) transcriptional fusion were grown in LB containing 100 µM IPTG to induce LsrR expression, with shaking. When indicated, the signal molecule, AI-2 (DPD), was added at final concentrations of 48 and 144 µM. (D) The SDS-PAGE gel pattern of secreted flagella proteins was evaluated in the absence or presence of 100 µM IPTG and/or 144 µM DPD. The secreted proteins were recovered from cell-free spent culture media by TCA precipitation. (E) Western blot analysis was conducted on cell extracts prepared from P*cat*-*lsr* strains harboring pJH1 grown in LB or LB containing 100 µM IPTG and/or 144 µM DPD, with shaking. These strains express the SopB protein tagged with a HA-epitope (SopB-HA) from the normal chromosomal location. (F) Monolayer of HEp-2 epithelial cells were infected with wild-type (WT) *Salmonella*, WT *Salmonella* harboring pJH1, and P*cat*-*lsr Salmonella* harboring pJH1 in the presence or absence of 100 µM IPTG and/or 144 µM DPD. After the gentamicin treatment, the numbers of internalized bacteria were determined by plating the bacteria on LB agar following appropriate dilutions. Values represent the relative amount of the intracellular bacteria and have been standardized to the level of internalization of WT strain, which was set at 1.00. The values are the average and standard deviation from three independent experiments, each done in triplicate.

As LsrR is a repressor of an operon comprising AI-2 transporter, overexpression of LsrR turns off the QS system of *Salmonella* and results in impaired invasiveness with reduced expression of SPI-1 and flagella as described above. Those effects may be associated with AI-2 signaling and QS or be an artifact resulting from ectopic expression of LsrR without any relevance to QS. We reasoned that we could distinguish between these two possibilities by modulating the levels of AI-2 in the overexpression strain because the phospho-AI-2 binds to LsrR, inactivating it [13]. If LsrR regulates SPI-1 and flagella genes in response to QS, addition of exogenous AI-2 should result in derepression of those genes. This experiment is complicated by the fact that the strain overexpressing LsrR cannot import and phosphorylate AI-2 due to repression of the *lsr* operon and *lsrK* [25]. To circumvent this problem, the promoters of the *lsr* operon and the *lsrK* gene were replaced with the promoter of the chloramphenicol resistance gene (P*cat*) which is constitutively expressed [26], and not repressed by LsrR (Fig. 6A).

To test whether LsrR regulates SP1-1 and flagella genes in response to QS, we examined the expression of chromosomal *invF-lacZ* and *fliC-lacZ* fusions in P*cat-lsr* strains (containing P*cat-lsrA* and P*cat-lsrK*) harboring pJH1 that were grown in the presence or absence of IPTG or exogenous AI-2. As expected, overexpression of LsrR reduced expression levels of the *invF* and *fliC* genes (Fig. 6B and C). Exogenous AI-2 restored expression of both the *invF* and *fliC* genes in dose dependent manner (Fig. 6B and C). In agreement with the transcriptional changes, the decreased production of flagella and the SopB protein observed upon LsrR overexpression was restored upon the addition of AI-2 (Fig. 6D and E). In WT with backbone plasmid, however, exogenous AI-2 with or without IPTG did not affect the expression of both *invF* and *fliC* genes (Fig. S3A and B) and the production of SopB protein (Fig. S3C).

We also asked whether addition of AI-2 to the *Pcat-lsr Pcat-lsrK* strain could restore the defective invasiveness resulting from LsrR overexpression. Overexpression of LsrR inhibited invasion of both the wild-type and the P*cat-lsr* strains into epithelial cells (17% and 25% invasion relative to the wild-type strain, respectively; Fig. 6F). However, addition of exogenous AI-2 restored the invasion phenotype of the P*cat-lsr* strain, but not the wild-type strain (Fig. 6F). Taken together, these findings suggest that the LsrR-mediated QS signaling system modulates expression of SPI-1 and flagella, and consequently invasion of *Salmonella* into host cells.

## Discussion

QS systems have been proposed to play a role in virulence of several pathogens based on studies of *luxS* mutant phenotypes, including *Escherichia coli* O157:H7 [27], [28], *Streptococcus pyogenes* [4], [29], *Porphyromonas gingivalis* [30], *Neisseria meningitides* [31], and *Salmonella* Typhimurium [18]. However, because the *luxS* gene has also been proposed to have non-QS functions [22], it can be difficult to define a role for LuxS-mediated QS in bacterial pathogenesis. Indeed, although we have reported the role of LuxS/AI-2 QS in regulation of SPI-1 in *S.* Typhimurium [18], another report indicated that LuxS did not play a role in SPI-1 regulation [19].

In *S.* Typhimurium, the regulatory network for AI-2 signaling is comprised of an AI-2 transporter complex, LsrABCD, the LsrR repressor, and the LsrK signal kinase [13]. LsrR regulates AI-2 uptake by directly binding and repressing the promoter of the *lsr* operon [21]. Because LsrR is the only known transcription factor in QS in *Salmonella*, we hypothesized that LsrR might connect LuxS*/*AI-2-mediated QS to SPI-1 regulation. Here, we report that the LsrR protein can negatively regulate expression of SPI-1 and flagella genes, and impair *Salmonella*’s invasion into mammalian cells.

Consistent with previous report [32], the deletion of *lsrR* gene in wild-type *Salmonella* did not alter *invF* expression. However, the deletion of *lsrR* in the *luxS* mutant strain restored the reduced expression of the *invF* and *fliC* genes in the *luxS* mutant strain to the wild-type levels (Fig. 2A and Fig.5A). These results suggest that LsrR is involved in regulation of SPI-1 and flagella genes, in contrast to a recent proposal that the regulatory function of LsrR is limited to the *lsr* operon [32]. The *luxS* deletion mutant would produce constitutively active LsrR due to the lack of AI-2, which supports the idea that LsrR might link LuxS-mediated QS and SPI-1 regulation and allows us to reveal additional target of LsrR in *Salmonella* Typhimurium. Overexpression of LsrR in wild-type *Salmonella* reduced both the mRNA and protein levels of SPI-1 (Fig. 3) and flagella genes (Fig. 5A, B, and C). These results emphasize that LuxS-mediated QS controls SPI-1 and flagella expression with AI-2-regulated LsrR activity playing a pivotal role.

We tested whether LsrR might regulate expression of SPI-1 and flagella genes by directly binding to their promoters. However, electrophoretic mobility shift assays failed to reveal LsrR protein binding to DNA fragments carrying promoters of SPI-1 and flagella genes such as *invF, sopB*, *flhDC,* and *fliC*, while LsrR did bind to the promoter DNA of *lsr* operon (data not shown). It is possible that additional factor(s) may be required for LsrR binding to the target promoter or that LsrR works indirectly. Although we do not understand the molecular basis of how the LsrR protein negatively controls transcription of SPI-1 and flagella genes, our findings clearly indicate that LsrR has regulatory targets than the *lsr* operon in *Salmonella*, as reported in *Escherichia coli* [25].

Overproduction of LsrR from a plasmid reduced entry into epithelial cells (Fig. 4), perhaps by generating increased levels of the active form of LsrR (i.e., LsrR not associated with phospho-AI-2). This result is consistent with our previous report that the *luxS* mutant was defective for invasion [18]. Moreover, decreased expression of flagella genes in bacterial cells possessing active form of LsrR has also been reported in a *E. coli luxS* mutant [7]. It is still possible that the altered expression of SPI-1 and flagella genes and reduced invasiveness resulting from LsrR overexpression might be artifacts unrelated with QS. However, that possibility is made less likely by the finding that treatment with exogenous AI-2 restored the behaviors of the LsrR-overexpressing *Salmonella* (Fig. 6). Overall, the data are consistent with a role for LsrR in regulating SPI-1 and flagella in response to QS.

The LsrR regulator represses expression of the *lsr* operon and the *lsrK* gene [25]. Consistent with this, exogenous AI-2 only counteracted the effects of LsrR overexpression in the engineered *Salmonella* strain (P*cat-lsr*) in which the promoters of the *lsr* operon and *lsrK* were replaced with the constitutive P*cat* promoter. To further verify whether the regulatory functions LsrR on SPI-1 and flagella genes are controlled by phospho-AI-2, we constructed a *Salmonella* strain (P*cat-lsrA*) carrying only *Pcat-lsrA* and not *Pcat-lsrK* (Fig. S4A). We reasoned that the P*cat-lsrA* strain could uptake AI-2 but not phosphorylate it due to LsrR-mediated repression of the *lsrK* gene which encodes the AI-2 kinase [1], [25]. The P*cat-lsrA* strain failed to restore expression of the *invF* and *fliC* genes upon the addition of AI-2 (Fig. S4B), indicating that LsrK is necessary for the LsrR protein to respond to AI-2 and control expression of SPI-1 and flagella (see Fig. S1).

When ingested by a host animal, *Salmonella* reach the small intestine and penetrate the epithelial cells. LuxS is a highly conserved protein, and many species of bacteria produce AI-2 [5], [11]. Based on our findings, we propose that *Salmonella* could recognize the site of infection by detecting and internalizing the AI-2 signaling molecules produced by microorganisms in the normal gut flora in addition to recognizing well known environmental signals such as high osmolarity to induce the expression of SPI-1 [33]. Moreover, studies of the known QS signaling mechanisms employing AI-2 have revealed that most QS bacteria are sensing the extracellular AI-2 rather than importing it [9], [11]. Thus, AI-2 molecules produced by normal flora would accumulate in the intestinal tract. The ability of *Salmonella* to consume AI-2 molecules could be beneficial for their virulence because the consumption of AI-2 could interfere with communication between other bacterial species and perhaps disrupt the flora in a way that is favorable for *Salmonella* pathogenicity.

In summary, we have presented evidence that active LsrR can negatively regulate expression of SPI-1 as well as flagella genes, and in doing so, reduce the ability of *Salmonella* to invade host cells. The effects of LsrR overexpression were relieved by exogenous treatment with the QS signal molecule, AI-2. These findings suggest that *S.* Typhimurium may require QS for successful invasion when they have reached the proper environment. QS may also enable *Salmonella* cells to modulate expression of virulence factors when they reached a sufficient population level in the right niche.

## Materials and Methods

### Ethics Statement

This study was carried out according to the recommended protocol for the care and use of laboratory animals from the Institute of Laboratory Animal Resource in Seoul National University, which is based on the Korean Animal Protection Law and Korea Food and Drug Administration regulation on the laboratory animals. The protocol was approved by the Committee on the Ethics of animal experiments of Seoul National University (Institutional Animal Care and Use Committee permit number: SNU-090717-3).

### Bacterial Strains, Plasmids and Growth Conditions

Bacterial strains and plasmids used in this study are listed in Table 1. *Salmonella enterica* serovar Typhimurium strains used in this study are derived from strain SL1344. Phage P22-mediated transductions were performed as described [34], [35]. All *Salmonella* strains were grown with (aerobically) or without shaking (anaerobically) at 37°C in Luria-Bertani broth (LB). For shaking cultures, 1.5 ml of media for glass tubes or 50 ml of media for flasks were used. For static cultures, 50 ml of media in sealed tubes were used. Antibiotics were used in the following concentrations; ampicillin 50 µg/ml, chloramphenicol 25 µg/ml, kanamycin 50 µg/ml.

**Table 1:** Bacterial strains and plasmids.

strain	Genotype	Source
*Salmonella typhimurium* strains		
SL1344	wild-type, Sm^R^	[45]
SR3306	*ΔluxS*	[18]
SR3326	*ΔluxS*, pJJ2	[18]
SR4506	*ΔluxS*, pJJ13	This study
SR4507	*ΔluxS ΔinvF*	This study
SR4508	*ΔluxS ΔinvF*, pJJ2	This study
SR4511	*ΔluxS ΔinvF*, pJJ13	This study
SR4558	SL1344, pUHE21-2*lacI* ^q^	This study
SR4559	SL1344, pJH1	This study
SR4656	P*cat-lsrK,* P*cat-lsrA*, *ΔlsrR*, pJH1	This study
SR3390	P*_invF_*::*lacZ*	This study
SR3391	P*_invF_*::*lacZ*, *ΔlsrR*	This study
SR3392	P*_invF_*::*lacZ*, *ΔluxS*	This study
SR3393	P*_invF_*::*lacZ*, *ΔluxS ΔlsrR*	This study
SR3394	P*_invF_*::*lacZ*, pUHE21-2*lacI* ^q^	This study
SR3395	P*_invF_*::*lacZ,* pJH1	This study
SR4668	P*_invF_*::*lacZ,* P*cat-lsrK,* P*cat-lsrA*, *ΔlsrR*, pJH1	This study
SR4669	P*_invF_*::*lacZ,* P*cat-lsrA*, *ΔlsrR*, pJH1	This study
SR4550	P*_fliC_*::*lacZ*	This study
SR4551	P*_fliC_*::*lacZ, ΔlsrR*	This study
SR4575	P*_fliC_*::*lacZ, ΔluxS*	This study
SR4610	P*_fliC_*::*lacZ, ΔluxS ΔlsrR*	This study
SR4576	P*_fliC_*::*lacZ,* pJH1	This study
SR4652	P*_fliC_*::*lacZ,* P*cat-lsrA*, *ΔlsrR*, pJH1	This study
SR4653	P*_fliC_*::*lacZ,* P*cat-lsrK,* P*cat-lsrA*, *ΔlsrR*, pJH1	This study
SR3598	*sopB-*HA, pUHE21-2*lacI* ^q^	This study
SR3599	*sopB-*HA, pJH1	This study
SR4658	*sopB-*HA, P*cat-lsrK,* P*cat-lsrA*, *ΔlsrR*, pJH1	This study
Plasmids		
pKD46	Ap^R^ P_BAD_-*gam-beta-exo oriR101 repA101^ts^*	[36]
pKD3	Ap^R^ FRT Cm^R^ FRT PS1 PS4 *oriR6Kγ*	[36]
pKD13	Ap^R^ FRT Km^R^ FRT PS1 PS4 *oriR6Kγ*	[36]
pCP20	Ap^R^ Cm^R^ *cI*857 λP_R_ *flp oripSC101^ts^*	[36]
pCE70	Ap^R^ FRT *tnpR lacZY* ^+^ *oriR6Kγ*	[37]
pACYC184	Tet^R^ Cm^R^ p15A *ori*	[37]
pUHE21-2*lacI* ^q^	rep_pMB1_ Ap^R^ *lacI* ^q^	[39]
pJJ2	pACYC184::*luxS* Cm^R^	[18]
pJJ13	pACYC184::*invF* Cm^R^	This study
pJH1	pUHE21-2*lacI* ^q^::*lsrR* Ap^R^	This study

### Construction of Strains

The method of Datsenko and Wanner [36] was used for chromosomal gene deletion, chromosomal *lacZ* fusion, and epitope tagging. For construction of a *Salmonella* strain carrying a chromosomal *fliC-lacZ* fusion, the Kan^R^ cassette from plasmid pKD13 was amplified using primers fliC-lacZ-F and FliC-lacZ-R (see Table 2 for primer sequences) and the resulting polymerase chain reaction (PCR) products were introduced into the SL1344 strain containing the plasmid pKD46, followed by selection for *fliC*::Kan transformants. The Kan^R^ cassette was removed using plasmid pCP20 [36] leaving an FRT site for introduction of *lacZY* using pCE70 [37]. Other chromosomal *lacZ* fusions were constructed following these procedures, and deletion mutants or epitope tagging mutants were constructed similarly including removal of the Kan^R^ cassette.

**Table 2:** Primers for construction of strains and plasmids, and qRT-PCR.

Primer name	Oligonucleotide (5'→3')
**for bacterial strain construction**	
invF-F	CTG TTA CGA AAA AGC GAG AGT TAC TGG TTG GTT GGC TAT TTG TAG GCT GGA GCT GCT TCG
invF-R	AAA CGC CAT AGT CTT CTC CCA GCA TTC TCA TCG TGT TGC CAT TCC GGG GAT CCG TCG ACC
invF-lacZ-F	TCG CCG CGG AAA TTA TCA AAT ATT ATT CAA TTG GCA GAC AAA TGA TGT AGG CTG GAG CTG CTT CG
invF-lacZ-R	CGC GGC ACA TGC CAG CAC TCT GGC CAA AAG AAT ATG TGT CT AT TCC GGG GAT CCG TCG ACC
sopB-HA-F	GCA GTC AGT AAA AGG CAT TTC TTC ATT AAT CAC ATC TTA TCC GTA TGA TGT TCC TGA TTA TGC TAG CCT CTA ATG ATG TAG GCT GGA GCT GCT TCG
sopB-HA-R	TAA ACG ATT TAA TAG ACT TTC CAT ATA GTT ACC TCA AGA CAT TCC GGG GAT CCG TCG ACC
fliC-lacZ-F	AGC CCA ATA ACA TCA AGT TGT AAT TGA TAA GGA AAA GAT CTG TAG GCT GGA GCT GCT TCG
fliC-lacZ-R	CCG CAC CCA GGT CAG AAC GTA ACG TGT CAA CCT GTG CCA AAT TCC GGG GAT CCG TCG ACC
Pcat-lsrK-F	GGG TAC AGA GTC GAG CCA TTT TTT TAT CCT CGG CTA TTT TCC CGG TAG TGA TCT TAT TTC ATT ATG GTG AAA GTT GGA ACC TCT TAC GTG CCG ATA TAT GAA TAT CCT CCT TAG TTC
Del_lsrR-F	GCA GCG GCG CGG GCA TAA TAC TTA CGC TAC AAG CGG CAT CAT ATG AAT ATC CTC CTT AGT TC
Pcat-lsrA-R	TAT TGT GAC TGA TTT GCA TGT TGC CTC CGC TCC CTC AAT GCC CGG TAG TGA TCT TAT TTC ATT ATG GTG AAA GTT GGA ACC TCT TAC GTG CCG ATT GTA GGC TGG AGC TGC TTC G
**for plasmid construction**	
invF-F2	GAG GCG CCA AGC TTT TAC ACA
invF-R2	AAT GTC CGC ATG CTA TCG TCT
lsrR-pF1	GTA AAG CCA GAA TTC GAC AAT GAG
lsrR-pR1	CGT TAC ATA GGA TCC TGT CAG TTA
**for real-time RT PCR**	
RT-sicA-F1	ATTTGGGATGCCGTTAGTGAAG
RT-sicA-R1	TAAACCGTCCATCATATCTTGAGG
RT-sopB-F1	AACCGTTCTGGGTAAACAAGAC
RT-sopB-R1	GGTCCGCTTTAACTTTGGCTAAC
RT-sopE-F1	CAACACACTTTCACCGAGGAAG
RT-sopE-R1	GGTCTGGCTGGCGTATGC
RT-invF-F1	GCAGGATTAGTGGACACGAC
RT-invF-R1	TTTACGATCTTGCCAAATAGCG
RT-flhD-F1	CAACGAAGAGATGGCAAACA
RT-flhD-R1	GACGCGTTGAAAGCATGATA
RT-fliA-F1	CCGCTGAAGGTGTAATGGAT
RT-fliA-R1	CCGCATTTAATAACCCGATG
RT-fliC-F1	AACGACGGTATCTCCATTGC
RT-fliC-R1	TACACGGTCGATTTCCTTCA
RT-rrsH-F1	CGGGGAGGAAGGTGTTGTG
RT-rrsH-R1	GAGCCCGGGGATTTCACATC

### Construction of Plasmids

An *invF* ^+^ plasmid for complementation was constructed as follows. The *invF* gene from SL1344 was amplified by PCR using primers invF-F2 and invF-R2, and the resulting product was cloned between HindIII and SphI sites of vector pACYC184 [38] to generate plasmid pJJ13. To construct the *lsrR* ^+^ plasmid pJH1 the *lsrR* gene from SL1344 was amplified by PCR using primers lsrR-pF1 and lsrR-pR1, and the resulting product was cloned between EcoRI and BanHI sites of vector pUHE21-2*lacI^q^* [39]. Primers used for plasmid construction are listed in Table. 2.

### β-galactosidase Assay

β-galactosidase assays were carried out in triplicate and enzyme activity (Miller units) was determined as described [40].

### Quantitative Real-time Reverse Transcription (RT)-PCR Analysis

*Salmonella* strains were grown in LB media aerobically or anaerobically to desired growth phase, and total RNA was isolated using RNaesy Mini Kit (Qiagen). After DNase treatment of the RNA solution, cDNA was synthesized using Omnitranscript Reverse Transcription reagents (Qiagen) and random hexamers. Quantification of cDNA was carried out using 2×iQ SYBR Green Supermix (Bio-Rad Laboratories), and real-time amplification of PCR products was performed using the iCycler iQ real-time detection system (Bio-Rad Laboratories). The relative amount of cDNA was calculated using a standard curve obtained from PCR using serially diluted genomic DNA as templates. The mRNA expression level of the target gene was normalized to the 16S rRNA expression level. Finally the values were normalized by those of wild-type. The sequences of the primers used are presented in Table 2.

### Secreted Protein Analysis by Sodium Dodecyl Sulfate-polyacrylamide Gel Electrophoresis (SDS-PAGE)

The proteins secreted into media were analyzed by SDS-PAGE. Bacterial overnight cultures were diluted (1∶100) in fresh LB and incubated aerobically or anaerobically. Cell-free spent media was obtained by centrifuging bacterial cultures (13,200 × *g* for 20 min), collecting the supernatant, and then removing bacterial cells from the supernatant by filtration (0.22-µm pore size, Millipore). Cell-free spent media were mixed with prechilled trichloroacetic acid (TCA) to final concentration of 10%, chilled on ice for 2 h, and then centrifuged at 15,000 × *g* for 15 min. The pellets were washed with acetone twice to remove all the TCA from the precipitates. After air drying, the pellets were dissolved in SDS sample buffer [41], and SDS-PAGE was performed using a 15% acrylamide gels from a mini-gel kit (Bio-Rad Laboratories, Inc). Proteins were visualized by staining with colloidal blue.

### Transmission Electron Microscopic Analysis

Bacterial cells grown in LB for 16 h at 37°C without shaking were deposited on carbon-film grids, partially dried, and stained with 2.0% uranyl acetate. The negatively stained samples were observed using a 2000EX transmission electron microscope (JEOL, Ltd) at an acceleration voltage of 100 kV.

### Motility Assay

Bacterial motility was measured as described previously [42]. Briefly, 0.3% LB agar plates were stab inoculated with 1 µl of a washed overnight culture. The plates were incubated upright at 37°C, and the diameter of bacterial growth was measured every hour. Where indicated, isopropyl-β-D-thiogalactoside (IPTG) was added to the LB agar at 100 µM.

### Western Blot Analysis

*Salmonella* strains expressing a HA epitope tagged version of SopB (SopB-HA) from the chromosome were grown in LB broth at 37°C. Bacteria were collected by centrifugation, and cell lysates were prepared using B-PER solution (Pierce). Cell lysates were resolved in 12% SDS-PAGE and the SopB protein was detected using anti-HA antibody (Sigma). Western blot was developed using anti-mouse immunoglobulin G horseradish peroxidase-linked antibody and the enhanced chemiluminescence detection system (Amersham Biosciences).

### Invasion Assay

HEp-2 cells (ATCC, CCL-23) were grown in Dulbecco’s modified Eagle medium (DMEM; Invitrogen) supplemented with 10% fetal bovine serum (FBS), penicillin (50 U/ml), and streptomycin (50 U/ml). Confluent monolayers for infection with bacteria were prepared in 24-well tissue culture plates. Each well was seeded with 2×10^5^ cells suspended in DMEM-10% FBS without antibiotics and incubated for 1 h at 37°C under 5% CO_2_. The wells were washed three times with phosphate-buffered saline (PBS) before bacterial cells were added. Bacterial cells were washed with PBS, suspended in pre-warmed DMEM medium, and then added onto the monolayer at a multiplicity of infection of 10∶1. After 1 h of incubation, the wells were washed three times with pre-warmed PBS to remove extracellular bacteria and then incubated for 1 h with pre-warmed medium supplemented with 100 µg/ml of gentamicin (Gm) to kill extracellular bacteria. The wells were then washed three times with PBS to remove the Gm. The HEp-2 cells were lysed in 1% Triton X-100 for 30 min, and then diluted with PBS. Dilutions of the lysed cell suspension were plated on LB agar medium to enumerate colony forming units (CFUs). As necessary, the 24-well tissue culture plates were subjected to low-speed centrifugation (10 min at 500 × *g*) [14]. This mild centrifugation promotes bacterium-epithelial cell contact, which bypasses the requirement for bacterial motility in *Salmonella* invasion. CFU values for mutant strains were divided by the mean CFU value for wild-type *Salmonella*.

### Animal Experiments

The experiment was conducted as described previously [43], [44]. Six-week-old female BALB/c mice were purchased from the Institute of Laboratory Animal Resources at Seoul National University. The mice were kept under pathogen-free conditions in filter-topped cages in Individually Ventilated Cage Racks (MSRS-M70S; Orient Bio Inc.) containing sterile bedding, and were fed sterile food and water ad libitum. All mice were acclimatized for at least 1 week prior to experimentation. Cohorts of ten mice were infected by oral gavage or intraperitoneally with about 10^7^ CFU or 10^3^ CFU of *Salmonella* cells in 100 µL of PBS, respectively. Water and food were withdrawn 4 h before infection, and re-supplied 2 h post-infection. Mouse mortality was recorded daily.

## Supporting Information

Figure S1 **Schematic diagram of LsrR-mediated QS regulatory circuit in** ***Salmonella typhimurium*** **.** AI-2 is synthesized by LuxS and accumulates extracellularly. Lsr transporter, encoded by *lsr* operon, internalizes the AI-2 into the cytoplasm, where it is phosphorylated to produce phospho-AI-2 by LsrK. LsrR represses the expression of *lsr* operon in the absence of phospho-AI-2, while it is de-repressed in the presence of phospho-AI-2 molecules that bind and inactivate LsrR [13]. In this study, we demonstrated that LsrR negatively controls the expression of SPI-1 and flagella genes and this regulation was abolished by phospho-AI-2.
(TIF)

Figure S2 **LsrR is required for the regulation of** ***invF*** **expression by LuxS.** Wild-type (WT) and other mutant strains carrying *invF-lacZ* fusion on chromosome were diluted in LB medium and grown with shaking, and β-galactosidase activity (Miller units) was determined at 4 h. If necessary, *lsrR* expression was modulated by adding IPTG as the indicated concentrations. Values shown are the means and standard deviation of three independent experiments.
(TIF)

Figure S3 **Treatment of AI-2 molecule or IPTG does not affect the expression of SPI-1 and flagella genes.** (A and B) The wild-type (WT) strains harboring backbone plasmid (pUHE) carrying a chromosomal *invF-lacZ* (A) or *fliC-lacZ* (B) transcriptional fusion were grown in LB for 4 h with shaking. IPTG or AI-2 was added at final concentrations of 100 µM and 144 µM, respectively. (C) Western blot analysis was conducted with cell extracts prepared from wild-type (WT) strains harboring pUHE grown in LB or LB containing 100 µM IPTG and/or 144 µM DPD, with shaking for 4 h. These strains express the SopB protein tagged with a HA-epitope (SopB-HA) from the normal chromosomal location.
(TIF)

Figure S4 **LsrK is required to inhibit the regulatory function of LsrR.** (A) Schematic of the genomic context of an engineered *Salmonella* strain (P*cat-lsrA*). The constitutively expressed promoter, promoter of chloramphenicol resistant gene (P*cat*), was used to substitute the original promoters of *lsrA.* (B and C) The engineered strains (P*cat*-*lsrA*) carrying a chromosomal *invF-lacZ* (B) or *fliC-lacZ* (C) transcriptional fusion harboring pJH1 were grown in LB for 4 h with shaking. To induce the production of LsrR from the *lac-*promoter, 100 µM of IPTG was supplemented to LB. If necessary, the signal molecule, AI-2 (DPD), was added at the final concentrations of 48 and 144 µM.
(TIF)
