# Supplementary figures and images for: LsrR-Mediated Quorum Sensing Controls Invasiveness of Salmonella typhimurium by Regulating SPI-1 and Flagella Genes
Source: PLoS One. 2012 May 18;7(5):e37059. doi: 10.1371/journal.pone.0037059 (PMC3356404; doi:10.1371/journal.pone.0037059)

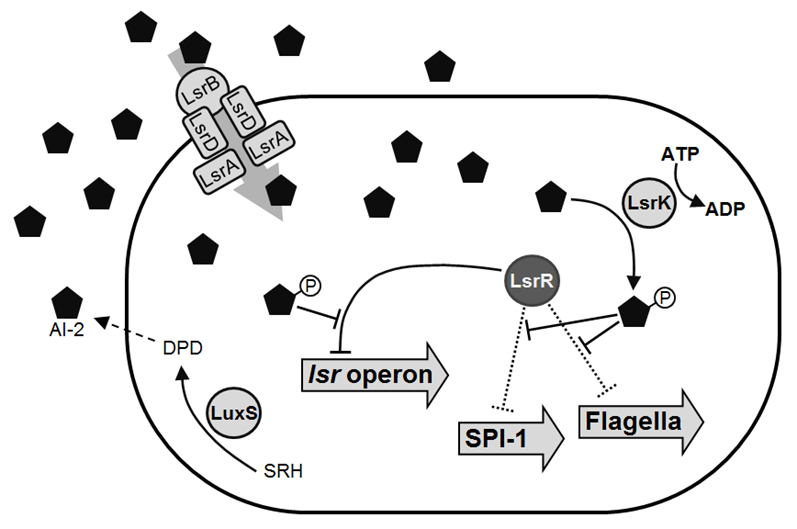

Supplement: Figure S1 — Schematic diagram of LsrR-mediated QS regulatory circuit in Salmonella typhimurium . AI-2 is synthesized by LuxS and accumulates extracellularly. Lsr transporter, encoded by lsr operon, internalizes the AI-2 into the cytoplasm, where it is phosphorylated to produce phospho-AI-2 by LsrK. LsrR represses the expression of lsr operon in the absence of phospho-AI-2, while it is de-repressed in the presence of phospho-AI-2 molecules that bind and inactivate LsrR [13]. In this study, we demonstrated that LsrR negatively controls the expression of SPI-1 and flagella genes and this regulation was abolished by phospho-AI-2. (TIF) [file pone.0037059.s001.tif]

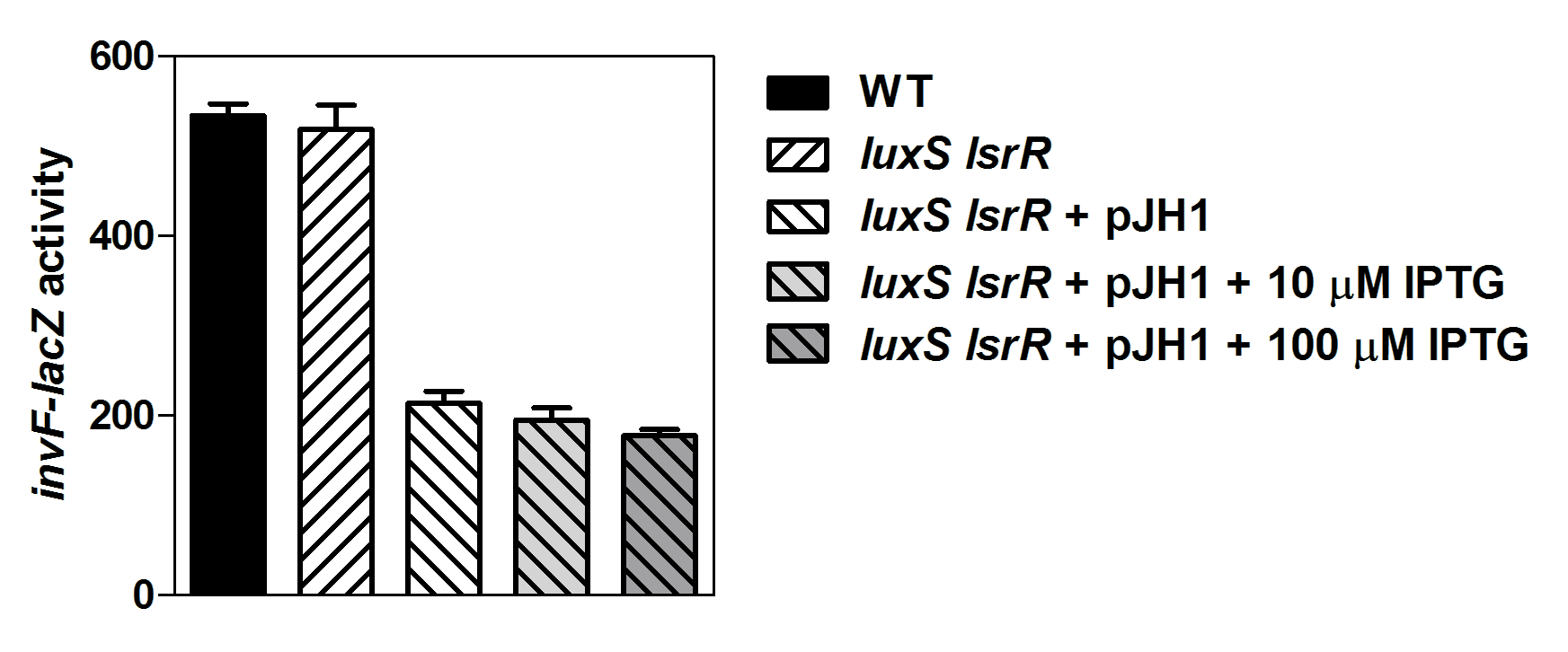

Supplement: Figure S2 — LsrR is required for the regulation of invF expression by LuxS. Wild-type (WT) and other mutant strains carrying invF-lacZ fusion on chromosome were diluted in LB medium and grown with shaking, and β-galactosidase activity (Miller units) was determined at 4 h. If necessary, lsrR expression was modulated by adding IPTG as the indicated concentrations. Values shown are the means and standard deviation of three independent experiments. (TIF) [file pone.0037059.s002.tif]

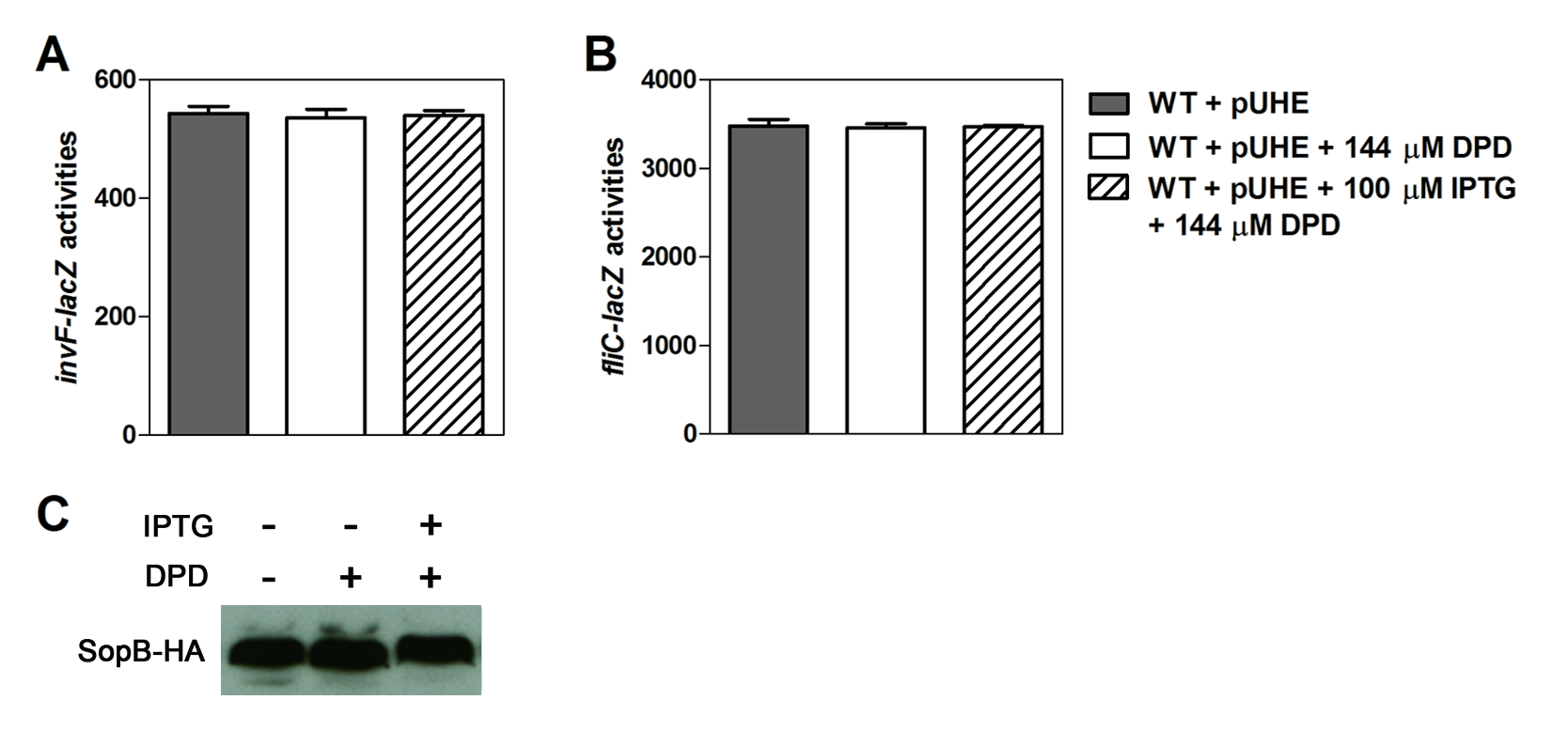

Supplement: Figure S3 — Treatment of AI-2 molecule or IPTG does not affect the expression of SPI-1 and flagella genes. (A and B) The wild-type (WT) strains harboring backbone plasmid (pUHE) carrying a chromosomal invF-lacZ (A) or fliC-lacZ (B) transcriptional fusion were grown in LB for 4 h with shaking. IPTG or AI-2 was added at final concentrations of 100 µM and 144 µM, respectively. (C) Western blot analysis was conducted with cell extracts prepared from wild-type (WT) strains harboring pUHE grown in LB or LB containing 100 µM IPTG and/or 144 µM DPD, with shaking for 4 h. These strains express the SopB protein tagged with a HA-epitope (SopB-HA) from the normal chromosomal location. (TIF) [file pone.0037059.s003.tif]

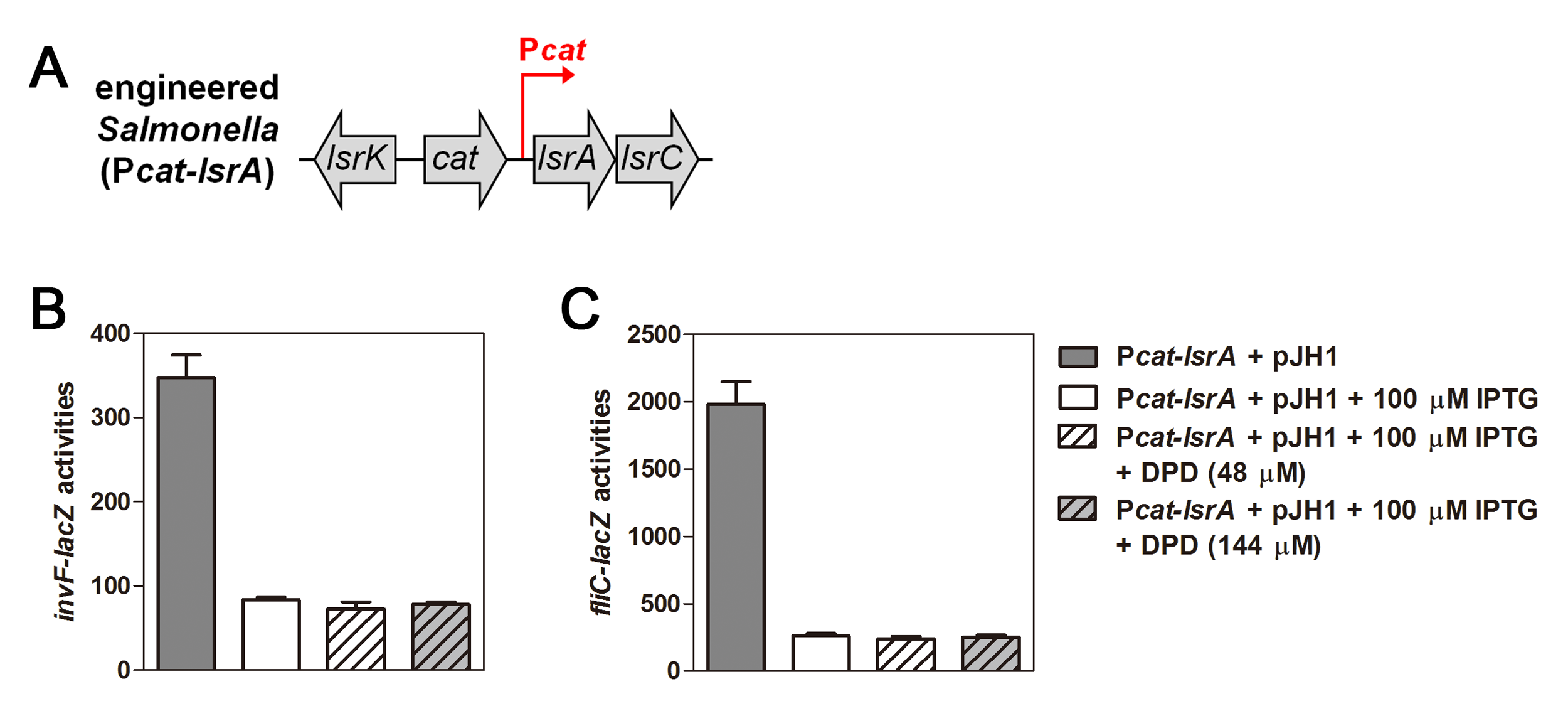

Supplement: Figure S4 — LsrK is required to inhibit the regulatory function of LsrR. (A) Schematic of the genomic context of an engineered Salmonella strain (Pcat-lsrA). The constitutively expressed promoter, promoter of chloramphenicol resistant gene (Pcat), was used to substitute the original promoters of lsrA. (B and C) The engineered strains (Pcat-lsrA) carrying a chromosomal invF-lacZ (B) or fliC-lacZ (C) transcriptional fusion harboring pJH1 were grown in LB for 4 h with shaking. To induce the production of LsrR from the lac-promoter, 100 µM of IPTG was supplemented to LB. If necessary, the signal molecule, AI-2 (DPD), was added at the final concentrations of 48 and 144 µM. (TIF) [file pone.0037059.s004.tif]
